# Placental quantitative susceptibility mapping and T2* characteristics for predicting birth weight in healthy and high-risk pregnancies

**DOI:** 10.1186/s41747-025-00565-2

**Published:** 2025-02-18

**Authors:** Morteza Pishghadam, Lylach Haizler-Cohen, Julius S. Ngwa, Wu Yao, Kushal Kapse, Sara N. Iqbal, Catherine Limperopoulos, Nickie N. Andescavage

**Affiliations:** 1 Developing Brain Institute, Division of Diagnostic Imaging and Radiology, Children’s National Hospital, Washington, DC USA; 2 Division of Maternal Fetal Medicine, Department of Obstetrics and Gynecology, MedStar Washington Hospital Center, Washington, DC USA; 3 Department of Radiology, School of Medicine, and Health Sciences, George Washington University, Washington, DC USA; 4 Division of Neonatology, Children’s National Hospital, Washington, DC USA; 5 Department of Pediatrics, School of Medicine, and Health Sciences, George Washington University, Washington, DC USA; 6 Present Address: Department of Pediatrics, School of Medicine, and Health Sciences, George Washington University, Washington, DC USA

**Keywords:** Birth weight, Fetal growth retardation, Magnetic resonance imaging, Placenta, Pregnancy (high-risk)

## Abstract

**Background:**

The human placenta is critical in supporting fetal development, and placental dysfunction may compromise maternal-fetal health. Early detection of placental dysfunction remains challenging due to the lack of reliable biomarkers. This study compares placental quantitative susceptibility mapping and T2* values between healthy and high-risk pregnancies and investigates their association with maternal and fetal parameters and their ability to predict birth weight (BW).

**Methods:**

A total of 105 pregnant individuals were included: 68 healthy controls and 37 high-risk due to fetal growth restriction (FGR), chronic or gestational hypertension, and pre-eclampsia. Placental magnetic resonance imaging data were collected using a three-dimensional multi-echo radiofrequency-spoiled gradient-echo, and mean susceptibility and T2* values were calculated. To analyze associations and estimate BW, we employed linear regression and regression forest models.

**Results:**

No significant differences were found in susceptibility between high-risk pregnancies and controls (*p* = 0.928). T2* values were significantly lower in high-risk pregnancies (*p* = 0.013), particularly in pre-eclampsia and FGR, emerging as a predictor of BW. The regression forest model showed placental T2* as a promising mode for BW estimation.

**Conclusion:**

Our findings underscore the potential of mean placental T2* as a more sensitive marker for detecting placental dysfunction in high-risk pregnancies than mean placental susceptibility. Moreover, the high-risk status emerged as a significant predictor of BW. These results call for further research with larger and more diverse populations to validate these findings and enhance prediction models for improved pregnancy management.

**Relevance statement:**

This study highlights the potential of placental T2* magnetic resonance imaging measurements as reliable indicators for detecting placental dysfunction in high-risk pregnancies, aiding in improved prenatal care and birth weight prediction.

**Key Points:**

- Placental dysfunction in high-risk pregnancies is evaluated using MRI T2* values.
- Lower T2* values significantly correlate with pre-eclampsia and fetal growth restriction.
- T2* MRI may predict birth weight, enhancing prenatal care outcomes.

**Graphical Abstract:**

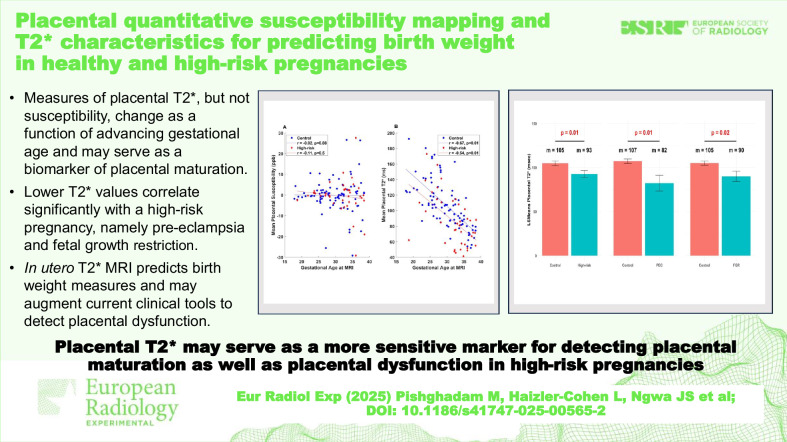

## Background

The human placenta plays a vital role in maintaining pregnancy health by delivering nutrients and oxygen to the developing fetus. Altered placental function, manifested by changes in oxygenation and blood flow, may lead to short and long-term complications, including hypertensive disorders of pregnancy, fetal growth restriction (FGR), stillbirth, preterm birth, perinatal mortality, and long-term neurodevelopmental morbidity in offspring [1–3]. Prenatal evaluation of the *in vivo* placenta may play a vital role in identifying and monitoring complicated pregnancies or pregnancies at risk for complications from placental dysfunction.

The fundamental obstacle to identifying placental dysfunction is the lack of early *in vivo* biomarkers of placental failure. Non-invasive modalities for the *in vivo* study of placental function are actively being developed. Current techniques, such as Doppler ultrasound of the umbilical artery, biophysical profiles, and non-stress tests, fail to provide direct measures of placental (and fetal) health and well-being; at best, these techniques offer surrogate measures lacking sensitivity and specificity.

Magnetic resonance imaging (MRI) is frequently used in pregnancy due to its established safety and superior tissue contrast over ultrasound. Qualitative MRI can be useful when ultrasound visualization is suboptimal (*e.g*., elevated body mass index) or its results are inconclusive (*e.g*., placenta accrete spectrum). Quantitative MRI can go beyond the traditional “lesion detection” MRI approach to serve as an early diagnostic marker to detect emerging placental dysfunction.

T2*-weighted imaging is an advanced quantitative MRI method for assessing the placenta in several high-risk conditions. Studies have shown its utility as a biomarker for placental dysfunction in various pregnancy complications, such as FGR, fetal congenital heart disease, and preterm pre-eclampsia (PEC). Lower T2* values are typically correlating with high-risk conditions [4–10]. Tissue magnetic susceptibility, inherent to all tissue classes, identifies specific biological tissue components with significantly varied susceptibility compared to water. Quantitative susceptibility mapping (QSM) is an emerging technique that directly quantifies susceptibility for studying tissue contents, with additional localization of regional susceptibility changes [11–13]. This additional sensitivity of QSM may provide distinct information regarding specific tissue components, enhancing our understanding of placental pathology beyond what can be collected from T2*-weighted imaging alone. The comparison of T2* weighted MRI and QSM in assessing placental function has not been previously studied.

The primary objective of this study was to compare the placental susceptibility and T2* measures between healthy and high-risk pregnancies that are associated with placental insufficiency, namely chronic hypertension (CHTN), gestational hypertension (GHTN), PEC, and FGR. We also sought to investigate the relationship between the placenta’s mean susceptibility and T2* with maternal and fetal parameters, including gestational age (GA) at MRI, maternal age (MA) at MRI, and fetal sex. Lastly, we investigated the ability of *in vivo* placental T2* and mean placental susceptibility to predict neonatal outcomes, namely birth weight (BW).

## Methods

### Subjects

This study was approved by our institutional review board at the Children’s National Medical Center, Washington D.C., USA. Prior to trial enrollment, all study participants provided written informed consent. Participants were recruited from December 2017 to February 2022 as part of a prospective observational study. The study enrolled eligible pregnant people of all races and ethnic backgrounds. Inclusion criteria included MA ≥ 18 years and singleton pregnancies. Exclusion criteria included multiple gestations, fetuses with known chromosomal abnormalities or genetic syndromes, and individuals who did not meet standard criteria for MRI safety due to physical or psychological reasons (*e.g*., claustrophobia). Participants underwent placental MRI up to two time points in pregnancy: the second and/or third trimester. Twenty-one study participants underwent two prenatal MRI studies (19 healthy controls and two high-risk).

Subjects were categorized as follows: high-risk group and control group. The high-risk group was defined based on the presence of any of the following conditions as documented and diagnosed by the caring obstetrician: CHTN, GHTN, PEC, and FGR, using standard clinical criteria. CHTN was diagnosed when the above blood pressure thresholds were met prior to 20 weeks of gestation; GHTN was diagnosed when elevated blood pressures were measured at least 4 h without preceding diagnosis of CHTN. PEC was diagnosed when, in addition to the aforementioned blood pressure criteria, there was also evidence of preeclamptic symptoms, proteinuria, or other laboratory abnormalities, and FGR was diagnosed when the estimated fetal weight was below the 10th percentile.

Relevant clinical data, including GA at MRI, MA at MRI, pregnancy complications, fetal sex, BW, and GA at birth, were extracted from the medical records. Subjects were categorized as healthy controls or having a high-risk pregnancy. The control group included pregnant individuals with no significant medical history, pregnancy complications, or abnormal screening results throughout gestation. The high-risk group comprised those with hypertensive disorders of pregnancy, including CHTN, GHTN, and PEC, as well as those with non-syndromic FGR.

### Data acquisition and processing

Study participants underwent MRI without sedation or contrast. A three-dimensional multi-echo radiofrequency-spoiled gradient-echo (GRE) sequence was used to acquire images. In the readout and slice-encoding directions, flow compensation was achieved. Prospective respiratory gating was conducted using respiratory bellows, with a 30% acquisition window set at end-expiration. All GRE sequences were acquired under normal respirations. All scans were performed on a GE Discovery MR450 1.5-T scanner using an 8-channel cardiac coil. For three-dimensional GRE, imaging parameters were: field of view 36–38 cm; acquisition matrix size 256 × 128; slice thickness 3 mm; and flip angle 30°; repetition time 93–97 ms, 15–16 echoes with echo time ranging from 4 to 80 ms; pixel bandwidth ± 31.25 kHz. The total scan time with parallel sensitivity encoding (*R* = 2) was 6–8 min. For reference anatomical imaging, T2-weighted two-dimensional single-shot fast spin-echo imaging was acquired on the same slices, with the following scan parameters: repetition time 1,200 ms; echo time 160 ms; matrix size 256 × 192, field of view 36–42 cm; and slice thickness 3 mm.

**Fig. 1:**
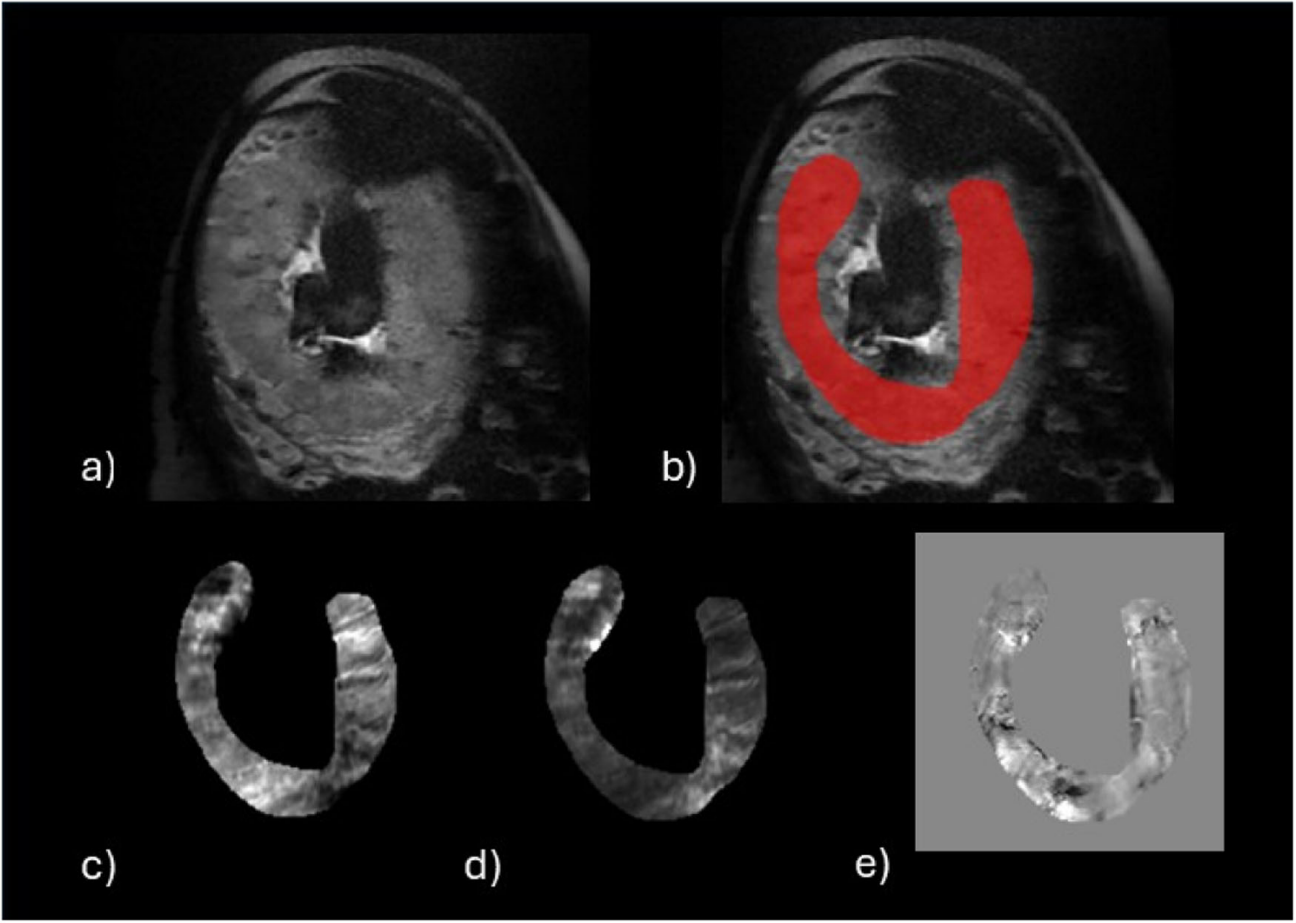
Example of placenta images and segmentation. **a** T2-weighted anatomical image of the placenta with (**b**) the corresponding segmentation mask in red, along with the corresponding R2* (**c**), T2* (**d**), and qsm images (**e**)

From GRE multi-echo data, the magnitude was rebuilt, and using ITK-SNAP software (version 3.8.0), the placenta was manually segmented on the first echo magnitude images of each slice using a corresponding T2-weighted anatomic image as reference. Precise segmentation was performed to avoid surface segments that could be contaminated by either the uterine wall or fetal tissue, as well as amniotic fluid. All segmentation was done manually by one of our team members (M.P., L.HC., and K.K.). An example of the placenta segmentation is given in Fig. 1. Thirty-three scans were randomly chosen and segmented by the alternate examiner to evaluate the interrater reliability. The intraclass correlation coefficient for placental segmentation was greater than 99%. The data was analyzed using the method developed by Zun et al [13]. The projection onto dipole fields method was utilized for background removal, while the morphology-enabled dipole inversion method was used for dipole inversion during the QSM reconstruction process [14–16]. T2* values were derived from GRE data through voxel-wise fitting of a monoexponential function, with noise-restricted voxels set to 400 [17]. The mean of susceptibilities and T2* for the entire placenta have been calculated. The mean placental susceptibility and T2* results are presented as part per billion (ppb) and milliseconds (ms).

### Statistical analysis

Descriptive and exploratory analyses were employed to summarize the baseline characteristics of our sample. Continuous variables were described using means and standard deviations, while categorical variables were presented as counts and percentages. Comparative analyses between the groups were performed using t-tests or Wilcoxon tests for continuous variables and Fisher’s exact tests for categorical variables. The Pearson correlation coefficient was calculated to assess the linear relationships between continuous variables. Least square means were calculated using the “emmeans” function to estimate the adjusted mean values of mean placental susceptibility and T2* across different groups while controlling for GA at MRI. Linear regression models were developed to assess the relationship between placental parameters and potential predictors, with adjustments made for confounders such as GA at MRI, MA at MRI, and fetal sex. The BW was standardized to BW *z*-scores, with consideration given to the sex and GA at birth [18].

We used univariate analysis to identify individual predictors associated with BW. The decision to include or exclude variables in the final model is guided by their *p*-values. A stringent *p*-value below 0.1 was used to include the predictive model. By selecting variables with *p*-values below the threshold, the model aims to capture the most relevant predictors while minimizing overfitting and ensuring the robustness of the final model. Therefore, using univariate analysis in this context facilitates the informed selection of predictors for inclusion in the predictive model, helping improve its accuracy and interpretability.

We employed a regression forest model for BW estimation, following the methodology used in the study by Dahdouh et al [19]. One thousand Learners regression trees were employed, using bagging with random predictor selections at each split, with the maximum number of decision splits equal to the number of observations minus one. This approach leverages the inherent robustness of the regression forest model against overfitting in high-dimensional settings, thereby negating the need for explicit feature selection. The model integrated key predictor variables identified through prior univariate analyses, ensuring the inclusion of relevant factors in the prediction process. The accuracy of BW estimates was evaluated using mean percent error, relative error, and the proportion of predictions within specified accuracy ranges (*e.g*., within 5%, 10%, and 20% of actual BW) [19]. All *p*-values were two-tailed, with *p* < 0.05 considered statistically significant, and confidence intervals were computed at a 95% confidence level. To verify the robustness and sensitivity of the results for multiple scans per subject, we also performed sensitivity analysis using single MRI measures as well with two additional models. All analyses were performed using MATLAB 2020b, MathWorks, Natick, MA, USA [20], and in R for Statistical Computing (version 4.3.2).

## Results

### Baseline characteristics of our study cohort

**Fig. 2:**
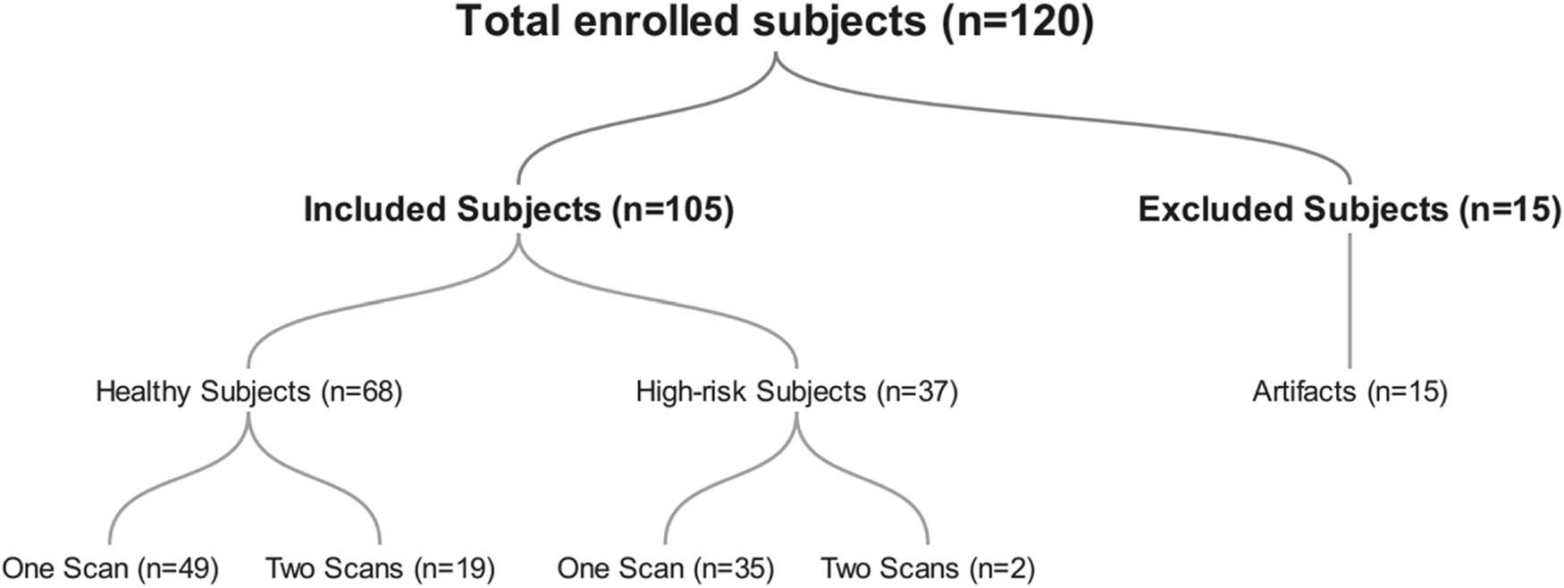
Flowchart of included subjects. The high-risk group included chronic hypertension (*n* = 8), gestational hypertension (*n* = 8), pre-eclampsia (*n* = 6), and fetal growth restriction (*n* = 15). One study participant with both fetal growth restriction and chronic hypertension was categorized under the fetal growth restriction group

A total of 120 participants underwent placental MRI. After the MRI data quality assessment, 15 examinations were excluded from the placental analysis because of severe artifacts. Ultimately, 105 subjects were included. A flowchart shows the selection of study subjects from the enrolled pregnant participants Fig. 2. The demographic and clinical characteristics of the 105 study participants are presented in Table 1. The study included 68 healthy control pregnant women and 37 high-risk pregnancies due to CHTN, GHTN, PEC, or FGR. One study participant with both FGR and CHTN was categorized under FGR. The mean GA at MRI for the entire cohort was 30.2 ± 5.2 weeks. The high-risk group had a higher mean GA at the time of MRI and lower GA at birth and BW compared to the control group. There was no significant difference in MA at MRI and fetal sex distribution between the two groups.

**Table 1:** Demographic and clinical characteristics of high-risk and healthy pregnant women

	Controls (*n* = 68)	High-risk (*n* = 37)	*p*-value
Gestational age at MRI (weeks)			
Mean (SD)	27.9 (4.6)	31.5 (5.1)	< 0.001
Maternal age at MRI (years)			
Mean (SD)	34.25 (4.7)	34.33 (5.5)	0.939
Fetal sex			
Male	30 (46.9%)	16 (43.2%)	0.299
Female	34 (53.1%)	17 (46.0%)	
Birth weight (grams)			
Mean (SD)	3,433.3 (463.23)	2,820.5 (643.2)	< 0.001
Gestational age at birth (weeks)			
Mean (SD)	39.5 (1.1)	38.1 (2.7)	< 0.001

Data are presented as mean (SD) or counts (%). Comparison of characteristics between groups by Fisher exact test for categorical variables and Student *t*-test for continuous variables

*SD* Standard deviation

Table 2 presents mean placental susceptibility and T2* across maternal and fetal parameters (GA at MRI, MA at MRI, and fetal sex) among healthy controls using linear regression models. There were no significant associations between maternal-fetal parameters and placental QSM. There was a significant negative association between the mean placental T2* and GA at MRI (*p* < 0.001), though MA and fetal sex were not significantly associated with placental T2*.

**Table 2:** Comparison of mean susceptibility and mean T2* measures of the placenta across covariates in adjusted estimates linear regression model in the healthy control group

	Mean susceptibility			Mean T2*		
Covariates	Estimate	Standard error	*p*-value	Estimate	Standard error	*p*-value
Gestational age at MRI	-0.02	0.17	0.889	-3.87	0.47	< 0.001
Maternal age at MRI	0.22	0.18	0.243	-0.96	0.51	0.063
Fetal sex	1.99	1.86	0.286	2.71	5.12	0.598

*MRI* Magnetic resonance imaging

### Placental QSM and T2* in healthy and high-risk pregnancies

**Fig. 3:**
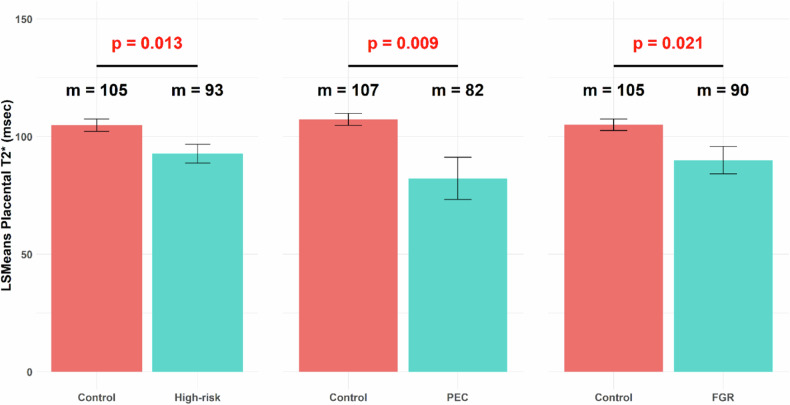
Bar plots comparing mean placental T2* after adjusting for gestational age at MRI of healthy control and high-risk groups. Left panel: healthy control (*n* = 87) and high-risk (*n* = 39) groups; middle panel healthy control (*n* = 87) and pre-eclampsia (PEC) (*n* = 7) groups; right panel: healthy control (*n* = 87) and fetal growth restriction (FGR) (*n* = 16) groups. MRI, Magnetic resonance imaging

After adjusting for GA at MRI, T2* values were lower in the high-risk group compared to controls (adjusted mean 93.0 ± 4.0 ms *versus* 105.0 ± 2.6 ms, *p* = 0.013; Fig. 3). A subgroup analysis revealed that the PEC and FGR groups had a significantly lower mean T2* compared to controls, while no differences were observed in the GHTN and CHTN groups (Fig. 3; Supplementary Table S1). We did not detect significant differences in mean placental susceptibility between control and overall high-risk groups (*p* = 0.928), nor in high-risk sub-groups when individually compared to healthy controls (Supplementary Table S1).

**Fig. 4:**
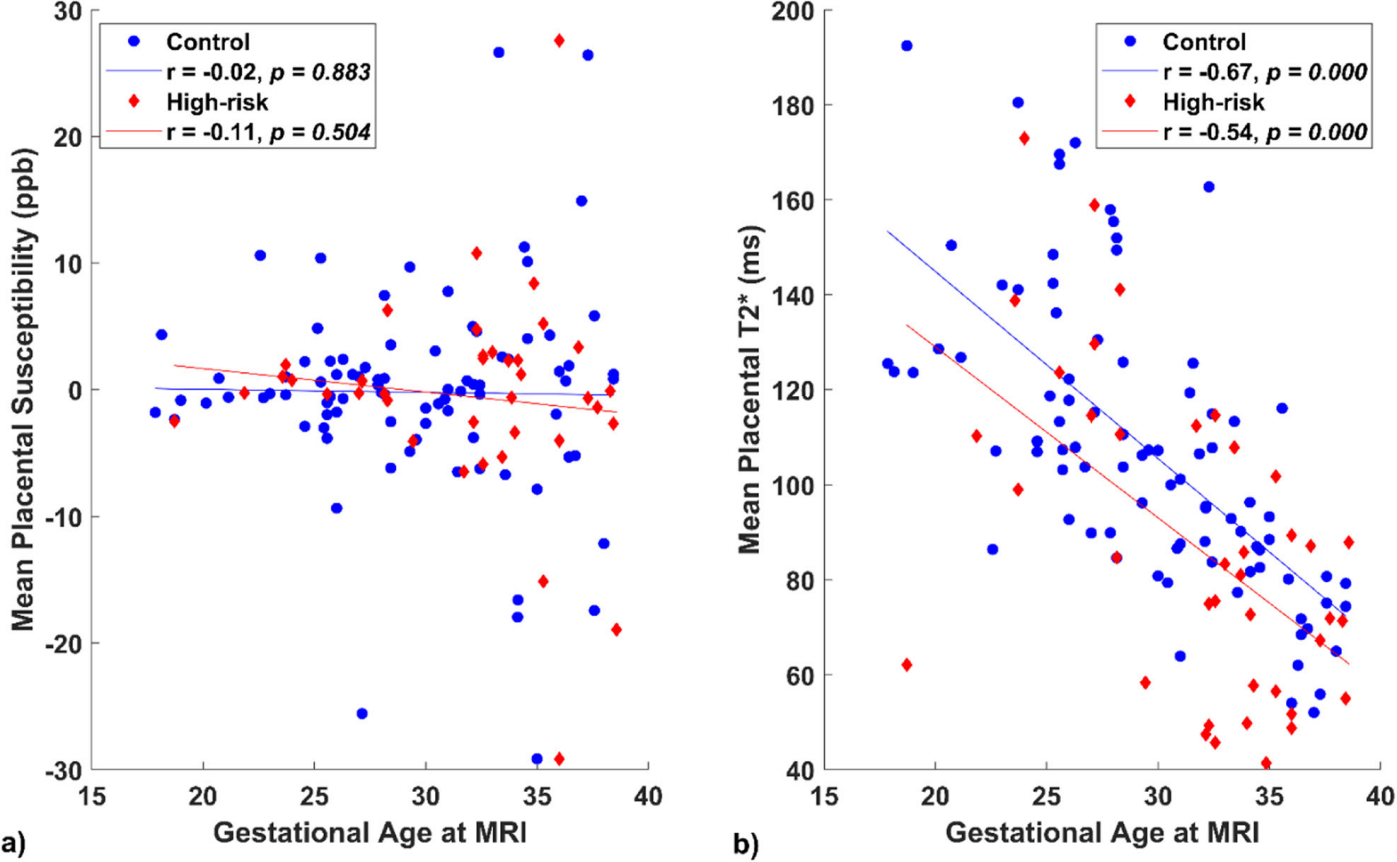
Correlations between placental parameters and gestational age at MRI across control and high-risk groups. **a** Association between mean placental susceptibility and gestational age at MRI. **b** Association between mean placental T2* and gestational age at MRI. Each dot or diamond represents a data point for control and high-risk groups. The blue and red lines in the figure represent the regression lines associated with the control and high-risk groups, respectively, highlighting trends. The Pearson correlation coefficient (*r*) is provided to quantify the strength and direction of the associations. ppb, Part per billion

We further explored the relationships between *in vivo* placental QSM and T2* measures and GA are presented in Fig. 4. We did not observe significant associations between mean placental susceptibility and GA at MRI in the control and high-risk groups (panel a); however, we did observe a significant negative association between mean placental T2* and GA at MRI in both the control and high-risk groups (*r* = -0.67, *p* < 0.001 and *r* = -0.54, *p* < 0.001, respectively, panel b).

### Placental QSM and T2* and pregnancy outcomes

Placental QSM and T2* relative to infant BW *z*-score are presented in Table 3. MA, placental T2*, and high-risk status were associated with BW *z*-score at delivery. Mean placental susceptibility did not have a statistically significant effect on birth weight *z*-score. An adjusted *R*^2^ value of 25% was observed.

**Table 3:** Regression analysis of factors influencing birth weight *z*-score for the entire cohort

Coefficients	Estimate	Standard error	*t*-value	*p*-value
Intercept	-6.20	8.87	-0.70	0.485
Maternal age at MRI	0.27	0.12	2.32	0.022
Gestational age at MRI	-0.28	0.17	-1.67	0.098
Mean placental T2*	0.08	0.03	3.23	0.001
Mean placental susceptibility	-0.08	0.07	-1.18	0.240
High-risk status	3.54	1.33	2.66	0.009

The birth weight was standardized to birth weight *z*-scores, with consideration given to the fetal sex and gestational age at birth. Adjusted *R*-squared: 0.25

*MRI* Magnetic resonance imaging

We then explored the potential for regression forest models of QSM and T2* in the determination of BW estimates using the significant variables detected above. Model 1 reflects *in vivo* placental T2* values relative to BW outcomes to serve as a reference. Model 2 integrates additional factors known to influence BW, namely GA at birth and fetal sex, while further adjusting T2* values by incorporating GA at MRI. There was a significant reduction in mean error for both health controls and high-risk pregnancies using Model 2 compared to Model 1. Further refinement in Model 3, by incorporating high-risk status, leads to improvements in error reduction with estimated BW for the control group of 3,380 ± 205 grams, compared to the actual BW of 3,420 ± 433 grams and estimated BW for the high-risk group of 2,902 ± 269 grams compared to the actual BW of 2,812 ± 537 grams. There was a tendency to underestimate BW in healthy controls (mean error = -39.50 grams) and overestimate BW in high-risk pregnancies (mean error = 89.6 grams). There was an improvement in the mean error percentage from model 1 to model 3 for both the control and high-risk groups (73.9% and 68.7%, respectively; Table 4).

**Table 4:** Accuracy of birth weight estimation models in control and high-risk groups

	Controls			High-risk		
Regression forest model	MPE ± RE	PPB (5 10 20) %	Birth weight mean error (grams)	MPE ± RE	PPB (5 10 20) %	Mean error (grams)
Model 1: Mean placental T2*	2.9 ± 14.6	(20 46 84)	-151.2	-26.4 ± 45.4	(31 51 68)	286.6
Model 2: Model 1 + GAB + Sex + GA_MRI_	1.5 ± 11.7	(29 63 88)	-96.9	-21.1 ± 37.8	(26 40 81)	217.8
Model 3: Model 2 + High-risk status	-0.3 ± 11.8	(27 61 91)	-39.5	-16.9 ± 37.9	(24 45 86)	89.6

The mean percentage error (MPE) computes the systematic deviation from the actual BW. The random error (RE) is the standard deviation of the error in percentage. PPB indicates the proportion of predicted birth value within 5%, 10%, and 20% of the actual BW

*GAB* Gestational age at birth, *GAMRI* Gestational age at magnetic resonance imaging

The study cohort includes subjects with MRI data available at one or two distinct time points. A notable subset of this cohort, comprising 21 subjects, had MRI data available from two separate time points. The sensitivity analyses included using single MRI measures with two additional models: Supplemental model 1 utilized single scans for all subjects, selecting the first available scan for those with two scans. Supplemental model 2 also utilized single scans for all subjects, selecting the second available scan for those with two scans. The results were consistent across all models (both models of single scans as well as the inclusion of multiple measures; Supplemental Table 2), affirming that utilizing all available data was a suitable approach for analysis.

## Discussion

This study revealed no significant difference in placental susceptibility between high-risk and healthy pregnancies. However, placental T2* values were significantly lower in the high-risk group, particularly within the PEC and FGR sub-groups. While existing literature suggests similarities between T2* and susceptibility maps, the potential sensitivity of T2* to both oxygenation and flow variations makes it a potentially more informative marker for placental function than susceptibility’s focus solely on oxygenation [21]. Our findings suggest that mean placental T2* might be a more sensitive marker than mean placental susceptibility for identifying placental dysfunction in high-risk pregnancies. We further demonstrate that placental T2*, GA at MRI, fetal sex, GA at birth, and high-risk status may also serve as a predictor of BW for both healthy and high-risk pregnancies.

Previous research by Dellschaft et al [21] suggests a significant increase in placental susceptibility to PEC, whereas our findings did not reveal a significant difference. This discrepancy may be attributed to several methodological differences. First, region of interest selection: We calculated susceptibility based on the entire placenta, while they used a smaller region of interest encompassing only four slices. This difference in the region of interest could potentially influence the average susceptibility value. Second, MRI acquisition and calculation methods: While both studies utilized MRI, there were differences in field strength (1.5 T *versus* 3 T); image acquisition and the calculation methods employed for susceptibility could contribute to variations in the final results. Notably, while magnetic susceptibility is generally considered independent of the field strength used for measurement [13], different measurement techniques and analysis methods can still introduce variations in the observed values. Our study utilized a lower magnetic field strength (1.5 T) compared to (3 T) [21]. Lastly, there were notable differences in subject selection and sample size. Our high-risk cohort reflected numerous high-risk conditions in addition to PEC., In exploring placental QSM within a healthy control cohort, we did not detect significant associations with GA at MRI, MA, or fetal sex, which suggests this modality may be limited as a potential biomarker of placental health over time. While this may reflect technical limitations in acquisitions or require more sophisticated post-processing analytics, additional studies are warranted to determine the utility of placental QSM in clinical practice.

Similar to previous studies, this study reveals a strong negative association between GA at MRI and mean T2* of the placenta [9, 10, 17, 22, 23]. These data suggest that T2* decreases as pregnancy progresses, likely reflecting normal placental maturation processes observed in healthy pregnancies. These processes involve morphological changes such as increasing villous density, fibrin deposition, and variations in oxygen consumption [6–8, 17, 24–30]. Accounting for differences related to advancing GA, this work also revealed lower placental T2* values in high-risk pregnancy conditions [4, 9, 10, 17, 31]. It is well established that both the quantity of hemoglobin and changes in the saturation of the hemoglobin molecule can be depicted as changes in the transverse relaxation time [4, 8, 10, 32] and vascular resistance, which also may be related to placental perfusion [4]. We propose that lower mean placental T2* detects known changes in placental structure and function, including abnormal morphology, placental fibrin deposition, and placental hypoxia.

Given the potential for T2* to serve as a biomarker of placental dysfunction and its ability to distinguish between healthy and high-risk pregnancies, we further demonstrated that T2* may augment pregnancy outcome prediction. Adjusting for GA at birth, GA at MRI, and fetal sex, T2* augmented prediction models of BW, with and without accounting for high-risk conditions. Previous studies have highlighted a strong correlation between mean placental T2* and BW [33, 34]. Similarly, other quantitative measures of placental MRI have been studied to improve the real-time detection of placental dysfunction, including radiomics, diffusion-weight imaging, and perfusion-related imaging [19, 35–40]. Several recent studies have demonstrated the discriminatory capabilities of both machine learning and radiomics to detect high-risk conditions like FGR [19, 35–37], and have demonstrated improved diagnostic accuracy when used in combination with ultrasound-based measures of the placenta [36]. Similarly, applications of diffusion-weight imaging in combination with ultrasound measures also report improved sensitivity and specificity in the accurate detection of FGR [27]. However, a recent study comparing T2* with intravoxel incoherent motion measures of diffusion-weighted placental imaging revealed that T2* was superior in identifying pathologic FGR compared to constitutionally small for gestation infants [41]. While collectively these studies suggest T2* may be superior to DWI or QSM, they also highlight how the optimal selection of MRI modalities, in isolation and combination, to improve the detection of placental dysfunction remains unknown. Further work is needed to improve the accuracy of placental insufficiency prenatally to better inform clinical practice, including delivery management and planning.

Although this work has strengths, several limitations should be considered. First, while the regression forest model achieved reasonable accuracy, the predicted results could be further improved using the model with larger and more diverse datasets. Second, our study considered individuals who underwent two separate scans at different time points in pregnancy as independent data entities. Although this approach may introduce bias in the data analysis, we implemented sensitivity analyses as a statistical approach to increase the validity of our findings.

In summary, our data suggest that T2* may be a more sensitive marker for detecting placental dysfunction than placental mean susceptibility. Machine learning approaches showed promising accuracy in BW estimation when integrating T2* into the model, with ongoing efforts to enhance accuracy. Increasingly, the application of advanced and quantitative MRI has revealed real-time assessments of placental development. These findings advance our understanding of placental function and its implications for pregnancy outcomes, highlighting the potential clinical relevance of mean placental T2* assessments in prenatal care. While these findings offer valuable insights, further research with larger and more diverse populations is needed prior to clinical implementation.

## Supplementary information

**Additional file 1: Supplementary Table 1:** Comparison of mean placental susceptibility and T2* between high-risk groups after adjusting for gestational age at MRI. **Supplementary Table 2:** Sensitivity Analysis of Mean Differences in Placental Susceptibility and T2* Values Between Healthy Control and High-risk Groups Across Various Scan Selection.

## Data Availability

The datasets used and/or analyzed during the current study are available from the corresponding author upon reasonable request.

## Abbreviations

BW: Birth weight
CHTN: Chronic hypertension
FGR: Fetal growth restriction
GA: Gestational age
GHTN: Gestational hypertension
GRE: Gradient-echo
MA: Maternal age
PEC: Preterm pre-eclampsia
QSM: Quantitative susceptibility mapping
