# Supplementary material for: Placental quantitative susceptibility mapping and T2* characteristics for predicting birth weight in healthy and high-risk pregnancies
Source: Eur Radiol Exp. 2025 Feb 18;9:18. doi: 10.1186/s41747-025-00565-2 (PMC11836258; doi:10.1186/s41747-025-00565-2)
Supplement: Supplementary file 1 — Additional file 1: Supplementary Table 1: Comparison of mean placental susceptibility and T2* between high-risk groups after adjusting for gestational age at MRI. Supplementary Table 2: Sensitivity Analysis of Mean Differences in Placental Susceptibility and T2* Values Between Healthy Control and High-risk Groups Across Various Scan Selection. [file 41747_2025_565_MOESM1_ESM.pdf]

Placental quantitative susceptibility mapping and T2* characteristics for predicting birth weight in healthy and high-risk pregnancies

ELECTRONIC SUPPLEMENTARY MATERIAL

Supplementary Table 1: Comparison of mean placental susceptibility and T2* between high-risk groups after adjusting for gestational age at MRI

	Parameters	N	Mean Susceptibility (ppb)	p	Mean T2* (ms)	p
Primary Analysis	Control	87	-0.25	0.928	105	**0.013**
	High-risk	39	-0.40		93	
Subgroup Analysis (Control vs. High-risk)	Control	87	-0.21	0.853	107	0.906
	CHTN	8	0.33		106	
	Control	87	-0.22	0.180	107	0.473
	GHTN	8	-4.43		101	
	Control	87	-0.20	0.181	107	**0.009**
	PEC	7	4.22		82	
	Control	87	-0.24	0.808	105	**0.021**
	FGR	16	-0.78		90	

Abbreviations: N: Number of examinations; ppb: part per billion; CHTN: Chronic Hypertension; GHTN: Gestational Hypertension; PEC: Pre-eclampsia; FGR: Fetal Growth Restriction. High-risk groups include CHTN, GHTN, PEC, and FGR.

Supplementary Table 2: Sensitivity Analysis of Mean Differences in Placental Susceptibility and T2* Values Between Healthy Control and High-risk Groups Across Various Scan Selection

	**Mean Susceptibility**			**Mean T2***		
	**Control**	**High-risk**	**P-value**	**Control**	**High-risk**	**P-value**
**All Scans**	N = 87	N = 39		N = 87	N = 39	
**Mean (SD)**	-0.21 (8.04)	-0.5 (8.41)	0.849	107.38 (30.55)	87 (33.2)	**0.001**
**Single Scan Model 1**	N = 68	N = 37		N = 68	N = 37	
**Mean (SD)**	0.65 (6.06)	-1.34 (7.2)	0.136	114.69 (29.44)	87.09 (34.1)	**<0.000**
**Single Scan Model 2**	N = 68	N = 37		N = 68	N = 37	
**Mean (SD)**	-0.25 (8.85)	-0.58 (8.63)	0.857	102.48 (28.91)	85.93 (33.73)	**0.009**

The primary analysis was conducted using the entire study cohort with all available scans. Sensitivity analysis employed two more models to analyze the cohort, each adopting a unique approach to scanned data. Model 1 focused on a single scan for each participant, utilizing only the first scan of subjects with two scans. Model 2, also used a single scan for each participant, considering the second scan from those with two scans. Collectively, these models offered a thorough and diverse analysis, encompassing various perspectives and scenarios.
